# A multi-method phenotypic study of sex differences in pragmatic language in autism

**DOI:** 10.3389/fpsyt.2026.1759436

**Published:** 2026-04-22

**Authors:** Emily Landau, Sarah E. Brooks, Janna Guilfoyle, Kritika Nayar, Stephanie Crawford, Jiayin Xing, Joseph C.Y. Lau, Gary E. Martin, Rob Voigt, Latha Valluripalli Soorya, Molly Losh

**Affiliations:** 1 Roxelyn and Richard Pepper Department of Communication Sciences and Disorders, Northwestern University, Evanston, IL, United States; 2 NYU Langone Health Hassenfeld Children’s Hospital, New York, NY, United States; 3 St. John’s University, New York, NY, United States; 4 University of California, Davis, Davis, CA, United States; 5 Rush University Medical Center, Chicago, IL, United States

**Keywords:** autism, autistic females, computational approaches, pragmatic language, sex differences

## Abstract

**Introduction:**

Autism spectrum disorder (ASD) is characterized in part by differences in pragmatic (i.e., social) language use. However, few studies on pragmatic language have included a meaningful number of autistic females, and even fewer have evaluated pragmatic language profiles for sex-specific differences. The existing literature on pragmatic language in autistic individuals without intellectual disability suggests that females may have stronger social communication skills compared to males, but findings are mixed, and there is not a clear profile of specific pragmatic skills that are liable to sex differences. It is also important to develop novel methodologies, such as computational methods, to characterize pragmatic language in ways less labor-intensive than gold-standard hand-coding methods, which are extremely time consuming and typically not feasible in clinical settings.

**Methods:**

The present study examined hand coding of pragmatic language data samples alongside multiple computational linguistic methodological approaches to characterize sex differences in pragmatic language in autistic males and females across narrative and semi-structured conversational tasks that might reveal context-specific patterns across sex and diagnostic groups.

**Results:**

Results indicated that most pragmatic domains differed between autistic and non-autistic groups, with autistic males showing the most obvious pragmatic differences, and that differences between diagnostic groups were more pronounced in the semi-structured conversational context. The alignment between computational and hand-coded findings was strongest in domains with clear theoretical overlap (e.g., frequency of emotional words) but was less consistent in areas that were less theoretically aligned (e.g., conversational dynamics and single word function).

**Discussion:**

These findings support the promise of computational methods for characterizing narrative abilities, though further study including validation against hand-coded approaches is warranted.

## Introduction

Research on autism spectrum disorder (ASD) has primarily been conducted on autistic males, owing to higher prevalence rates of ASD in males (i.e., recent CDC data indicating sex ratios ranging from 3:1 and 4:1; 1 2–8). However, recent research has raised the critical question of whether such prevalence rates have been influenced by historically male-defined diagnostic criteria derived from predominantly male samples, contributing to the under-identification of autistic females (3, 9, 10). Autistic females may also engage in masking, or compensatory behaviors aimed at concealing or minimizing autistic traits, which can impact the timing and accuracy of an autism diagnosis and contribute to under-identification (11–15). Understanding the female presentation of ASD is therefore key to supporting accurate diagnostics, supporting tailored interventions, and understanding the unique social, behavioral, and mental health considerations of autistic females (1, 3, 9, 10).

### Pragmatic language in autism

Autistic individuals show a wide range of pragmatic challenges, including those related to social responsiveness, initiating and maintaining conversational topics, contingent language, repairing communication breakdowns, producing communicative conversational cues, off-topic language, eye contact, gestures, and prosody (i.e., rate, rhythm, intonation of speech) (c.f., 16–38). Difficulties with pragmatic language constitute a key clinical characteristic of ASD and are part of the core diagnostic criteria, underscoring the importance of pragmatics in diagnostic considerations (39). Several studies have directly compared pragmatic language abilities across contexts, including conversational and narrative (i.e., storytelling) contexts, showing that unstructured, conversational tasks tend to elicit more differences compared with structured tasks, offering insight into patterns of language more reflective of everyday communication (19, 40–44).

### Sex differences in pragmatic language

A recent scoping review underscored the ongoing underrepresentation of autistic females in the pragmatic language literature (45). Studies frequently either exclude autistic females or aggregate their data with males, with limited power to detect sex-specific differences, precluding identification of female-specific language characteristics (45). In neurotypical individuals, evidence suggests that females exhibit stronger verbal and social communication skills than males, including larger vocabularies, more turn-taking during conversations, and greater use of language referring to emotions and other internal states (46–51). In autism, some evidence suggests a similar trend when IQ is controlled, with females showing stronger social communication skills than males. For example, females have been found to produce more cohesive storytelling, describe emotions more frequently, and demonstrate greater social reciprocity (52–57). Other studies, however, have reported mixed findings, with some finding no clear sex differences in structural or pragmatic language, particularly in adults with higher IQs or education levels, suggesting that differences may be more pronounced earlier in development or in specific contexts (58–68).

Among verbally fluent autistic individuals, evidence suggests that females may use more sophisticated conversational and storytelling strategies, including use of more figurative language (e.g., irony, rhetorical questions) and social words reflecting cognitive and emotional processes compared to males (69–72). These differences in social communication skills also translate to peer relationships; females with and without autism generally show greater friendship quality and reciprocity compared to males, although autistic participants of both sexes generally show more difficulty in friendships than their non-autistic peers (73). Importantly, some evidence suggests that females may display qualitatively distinct and more subtle pragmatic differences than autistic males, such as stronger use of figurative language (15, 55, 71, 74). However, it will be important to expand on these findings in larger samples and examining a greater variety of pragmatic skills.

Studying communication contexts that tap different pragmatic skills is critical as contextual demands have been revealed as a key factor in the pragmatic challenges in autism in general, and also appear to play a key role in revealing sex differences. In conversational settings, autistic females speak more and use more social words than autistic males, while males may speak more slowly and interrupt less frequently (70, 75). Assessments of pragmatic violations suggest a stepwise pattern, with autistic males showing the most frequent pragmatic violations, followed by autistic females, then non-autistic individuals (76). In narrative contexts, autistic females show fewer differences in story details, cognitive state language, and prosodic qualities than males (69, 71, 77, 78). These findings together suggest that pragmatic differences are most evident in communication contexts requiring greater social reciprocity (e.g., conversation versus structured narration). However, some studies report no sex differences, underscoring that these effects are not universal and may depend on sample characteristics or task type (79).

### Methodologies for assessing pragmatic language

Prior studies of pragmatic language in autism have traditionally applied detailed hand-coding schemes of operationally-defined pragmatic features. Whereas this approach has provided critical information on the pragmatic profile of autism, hand coding is time-intensive and requires substantial training and expertise to apply with strong reliability and validity. Computational and natural language processing methods have recently emerged as a promising alternative, allowing for larger sample sizes and greater clinical-translational potential (80, 81). These approaches include semantic similarity measures (e.g., Latent Semantic Analysis, word2vec) and word classification tools (e.g., Linguistic Inquiry and Word Count), which can capture discourse features such as coherence, off-topic language, use of cognitive or emotion-related words, and thematic patterns (c.f., 28, 69, 82–96).

Early computational linguistic work in ASD has revealed meaningful linguistic differences that align with findings from hand-coding studies, including poorer semantic cohesion, increased off-topic language, and repeated focus on restricted interests (28, 82, 87, 93, 94, 97). Recent methods, such as Next Sentence Prediction using transformer-based language models such as BERT, leverage contextual information across sentences to quantify conversational coherence, and have been shown to align with hand-coded measures of pragmatic violations (98).

Despite these advances, pragmatic language extends beyond individual words and sentences, encompassing prosody, context, and nonverbal cues, which are all features of complexity that remain difficult for current computational methods to fully capture. Hand-coding methods have been designed and validated specifically for detailed analyses of different verbal and nonverbal pragmatic language features, such as gesture, gaze coordination, prosody, and turn-taking. While computational approaches show considerable promise, additional work is needed to integrate them with hand-coding methods, refine their sensitivity for capturing a wide range of pragmatic features, and evaluate them across multiple discourse contexts. Importantly, sex differences in autism may be differentially expressed and obscured from detection from standard coding systems depending on the pragmatic features assessed and the methods used to measure them. In the autistic population more generally, highly structured storybooks appear to often mask underlying differences in narrative generation abilities that are more robustly evident in conversational contexts. Thus, methodological decisions concerning the context examined and method of capturing pragmatic skill differences are critical to consider in characterizing potential sex-linked patterns of pragmatic skills. This study addresses this need by applying hand-coding and computational methods to multiple pragmatic contexts, while also examining sex differences in autism, to examine how measurement approach influences the detection of sex differences in autism, and applying sensitive hand-coding methods to parallel to more novel and experimental computational approaches.

### Current study

This study aimed to characterize sex differences in pragmatic language in a cohort of age- and IQ-matched (based on group means, *p*s >.454) males and females with and without ASD by applying a multi-method approach across linguistic contexts capitalizing on both hand-coded and computationally-derived data. Computational measures were compared to hand-coded data to understand convergence across methods and sensitivity of computational methods for capturing key pragmatic features. It was predicted that both autistic males and females would demonstrate significantly poorer conversational and narrative pragmatic language skills compared to males and females without autism using both hand-coded and computationally-derived pragmatic language variables, and that autistic females would exhibit fewer differences than autistic males. More differences were predicted to emerge in the less structured conversational context relative to the narrative context. It was also predicted that conceptually related hand-coding and computational measures would be strongly correlated, as they are designed to capture overlapping aspects of pragmatic language. Examining this convergence allowed us to understand which pragmatic features computational tools can reliably capture, where they complement traditional hand-coding approaches, and where they may extend beyond traditional methodologies to identify subtle, large-scale, or cross-contextual language patterns, such as lexical diversity and semantic coherence. Of note, this study examined differences in pragmatics based on biological sex, owing to the focus on biological mechanisms influencing pragmatics in autism. Sociocultural factors related to gender identity are known to influence pragmatics, but are beyond the scope of the present study.

## Methods

### Participants

Participants included 73 autistic males, 27 autistic females, 40 non-autistic males, and 47 non-autistic females. Participants were recruited through several resources, including physicians’ offices, local clinics, parent support groups, community centers, schools, registries, and advocacy groups. All individuals in the study provided informed consent, and procedures were approved by the Northwestern University Institutional Review Board.

Demographic information is presented in **Table 1**. Participants were excluded if they reported a family history of genetic conditions related to ASD (e.g., fragile X syndrome, neurofibromatosis type 1). Participants with ASD had a previous clinical diagnosis of ASD and met criteria for ASD on the Autism Diagnostic Observation Schedule-Second Edition (ADOS-2; 99) and/or Autism Diagnostic Interview-Revised (ADI-R; 100). In non-autistic groups, participants were excluded if they met the threshold for ASD based on the ADOS-2, or if they had an atypical developmental history (e.g., significant speech delays). Participants were also excluded if they had a verbal IQ (VIQ) < 80 based on the Wechsler Abbreviated Scale of Intelligence (WASI) or the Wechsler Intelligence Scale for Children (WISC)—Third or Fourth Edition (101, 102) to ensure that participants were verbally fluent with intact structural language abilities. All participants were native English speakers. Participants were administered the ADOS-2 Module 3 or 4 depending on age. All participants were between the ages of 10 and 39 years of age. Ten was chosen as a cutoff for age because complex narrative and conversational abilities are typically well-established by this age (103–107).

**Table 1:** Demographic information of participants.

Variable	Autistic males	Autistic females	Non-autistic males	Non-autistic females
	M(SD) range	M(SD) range	M(SD) range	M(SD) range
N	73	27	40	47
Age (years)^b, c^	18.2 (6.3) 10.0-37.4	23.4 (8.1) 11.1-36.7	18.4 (7.2) 10.2-37.7	19.1 (5.3) 10.1-31.6
FSIQ^a, b, c^	104.6 (13.6) 74-135	110.7 (14.0) 83-131	119.5 (11.4) 91-135	116.7 (11.0) 88-142
VIQ^a, b, c^	105.3 (14.5) 81-146	111.5 (13.6) 86-131	119.4 (12.3) 93-140	119.0 (10.2) 98-142
PIQ^a^	103.3 (16.5) 53-135	108.9 (15.6) 73-131	117.0 (12.8) 87-141	112.0 (13.5) 79-143
Race	n (%)	n (%)	n (%)	n (%)
White	49 (67.1%)	23 (85.2%)	27 (67.5%)	32 (68.1%)
Black/AA	4 (5.5%)	1 (3.7%)	2 (5%)	2 (4.3%)
Asian	1 (1.4%)	2 (7.4%)	4 (10%)	10 (21.3%)
NH/PA	0 (0%)	0 (0%)	0 (0%)	0 (0%)
Biracial	2 (2.7%)	0 (0%)	3 (7.5%)	1 (2.1%)
Other	2 (2.7%)	0 (0%)	0 (0%)	1 (2.1%)
Not reported	15 (20.5%)	1 (3.7%)	4 (10%)	1 (2.1%)
Ethnicity	n (%)	n (%)	n (%)	n (%)
Hispanic	0 (0%)	1 (3.7%)	2 (5%)	0 (0%)
Non-Hispanic	14 (19.1%)	13 (48.1%)	15 (37.5%)	26 (55.3%)
Not reported	59 (80.9%)	14 (51.9%)	23 (57.5%)	21 (44.7%)

To maximize samples sizes for analyses within each task, all eligible participants were included, even when participants’ data were not available across all tasks. Therefore, numbers above represent the largest sample across all tasks, including all participants who contributed at least one task; FSIQ, Full Scale IQ; VIQ, Verbal IQ; PIQ, Performance IQ; Black/AA, Black/African American; NH/PA, Native Hawaiian/Pacific Islander.

^a^ Significant difference between autistic male and non-autistic male groups.

^b^ Significant difference between autistic male and female groups.

^c^ Significant difference between autistic female and non-autistic female groups.

### Narrative and conversation elicitation

#### Semi-structured conversation

The ADOS-2 (99) was used to elicit semi-structured conversations and has been applied extensively in studies of pragmatics in ASD and related neurodevelopmental disorders (c.f., 19, 29, 30, 70, 108, 109). All team members providing direct assessment with the ADOS-2 achieved reliability with, and were monitored and trained by, an ADOS-2 certified, research reliable team member.

#### Narrative task

Participants narrated a 24-page picture book, *Frog Where Are You* (110). This picture book is about a boy and his pet dog as they search for their missing frog, and it has been used extensively in the narrative literature (28, 42, 87, 103, 111–121). Methods of narrative elicitation were consistent with Lee et al. (87) and Nayar et al. (115), and involved narrating the story page-by-page while viewing the stimuli on a computer or eye tracker. There were no time limits for this task, and the examiner advanced the page once the participant indicated they had finished speaking. Of note, the sample of participants in the present study overlaps with the participants in Lee et al. (87), Nayar et al. (115), and Landau et al. (122). However, the prior studies did not examine sex differences nor have a large enough autistic female sample to do so (largest autistic female N in prior publications = 9). The current study is enriched for autistic females and has a larger sample size across diagnostic and sex groups compared to past publications.

#### Transcription

Language samples from the ADOS-2 (conversational tasks only) and the narrative task were transcribed from video and audio recordings using Systematic Analysis of Language Transcripts (SALT) conventions (123). For the ADOS-2, tasks with a narrative component (i.e., picture book, cartoons, create a story, demonstration task) were not transcribed, because the ADOS-2 was used in this study to elicit conversational samples. Transcribers were trained to >80% word reliability for each task. Sixteen percent of narrative files were randomly selected (N = 22) with even distribution across diagnostic and sex groups for transcription reliability, and mean word-level reliability was 95.55%. For the ADOS-2, 11.8% of files were randomly selected (N = 17), and the mean word-level reliability was 87.54%.

### Narrative coding systems

An overview of all coding systems and approach (hand coded vs computational) is presented in **Table 2**.

**Table 2:** Overview of coding systems and approach.

Coding system	Narrative	Conversation	Features coded
Hand Coded			
Narrative Quality	X		Narrative structure and evaluation
Pragmatic Rating Scale-School Age (PRS-SA)		X	Different types of pragmatic behaviors
Speech Acts	X	X	Functional categories of utterances
Speech Act Sequences	X	X	Patterns of speech acts
Computationally Coded			
Linguistic Inquiry Word Count (LIWC) Novel Dictionary	X	X	Functional categories of words
Speech Act Sequences	X	X	Sequential patterns of speech acts based on hand-coding

#### Narrative quality coding

The narrative coding scheme applied to story-telling samples is consistent with Nayar et al. (115). The coding scheme examines several key domains of narrative quality, such as the number of descriptions of characters’ thoughts and emotions, as well as the causal attributions of characters’ thoughts, emotions, and behaviors.

##### Story Structure

The presence of key story elements was coded across the narrative, including the setting, plot establishment, five search episodes (e.g., boy and dog searching for the frog), and resolution. Each present, key story element was tallied, and scores were summed to create a total score for *Story Components*. Possible scores ranged from 0 (no key story elements) to 8 (all 8 key story elements). This variable was also examined dichotomously (*Missing Story Components)* to capture if participants included all story elements or missed any elements.

##### Thematic Coherence

Descriptions of thematic establishment (i.e., missing frog and start of the search) and maintenance (i.e., continued search for the frog) of the story’s theme were coded as present or absent.

##### Evaluation

The ability to enrich stories with the narrator’s perspective of the characters’ internal states was assessed by examining descriptions of *Affect, Cognition*, and *Causal Explanations.* Descriptions were tallied and examined as a percent of word count to control for story length. *Affective States and Behaviors* included descriptions of the character’s affective states (e.g., happy, angry) and emotional behaviors related to affective states (e.g., smile, cower). *Cognitive States and Behaviors* included descriptions of characters’ cognitive states (e.g., know, think) and behaviors related to cognitive states (e.g., attack, hide). *Causal Explanations* included causal descriptions of behaviors, affect, and cognition. Causal explanations were coded for explicit language used to denote causality, such as “because”. More subtle uses of causal language were also included, such as language markers including “as a result”, “in order to”, and “therefore”.

##### Other Codes

Narratives were also evaluated for the presence of excessive detail and topic perseveration. Excessive detail was rated as excessive or not excessive. Topic perseveration was coded when there were three or more mentions of one topic that were tangentially related to the narrative throughout the story.

#### Narrative quality reliability

All transcripts were coded by raters blind to group status. Coders were trained to >80% training reliability on at least 3 files. Thirty-five percent of files were double coded for reliability. Reliability for categorical variables was assessed using percent agreement, and reliability for continuous variables was assessed using intraclass correlation coefficients (ICCS; (124). ICCs from .5-.75 represent moderate agreement, .75-.9 represent good agreement, and >.9 represents excellent agreement (125). Percent agreement for categorical variables met or exceeded 80% across groups, except for ratings of presence of setting in the ASD female group (62.5%). ICCs for continuous variables were greater than .84, indicating good to excellent agreement across codes.

#### Narrative speech acts coding

Each utterance was manually categorized using a dialog tag set developed for spontaneous spoken speech based on Carletta et al. (126). Speech acts were coded across transcripts using Praat software without audio or video input. Examiner utterances were excluded. Speech acts were examined as a percent of total utterances. In addition to examining each speech act type separately, the number of types of speech acts were examined to assess the diversity of speech acts across the 7 speech act categories.

Statements included any narrative-related utterances, such as utterances about the story.

Questions included any yes/no questions, wh- questions, or those marked by intonation (indicated in transcript).

Nonnarrative speech included off-topic, nonnarrative language (e.g., bathroom request).

Character speech included speech where the narrator took on the character’s voice as part of their narration, which was marked in the transcript.

Sound effects included utterances used to imitate sounds (e.g., “Splash!”).

Thinking language included descriptions of the participant’s own experience telling the narrative (not about the character’s experience) (e.g., “This is really hard for me”).

Incomplete utterance included a clear abandonment of an utterance or unintelligible utterances.

#### Narrative speech acts reliability

Files were coded by raters blind to group status. Coders were trained to >80% overall reliability on at least 3 files. Nineteen percent (N = 28) of files were double coded for reliability. Reliability was assessed using intraclass correlation coefficients (ICCS) (124). ICCs were greater than .61, indicating moderate to excellent agreement, except for sound effects, which had an ICC of .41.

### Conversational coding systems

#### Pragmatic Rating Scale- School Age

The Pragmatic Rating Scale-School Age (PRS-SA) (127), adapted from the Pragmatic Rating Scale (PRS) (128) is a pragmatic language rating system applied to videotaped conversational interactions during the ADOS-2, and has been used in studies of pragmatics in ASD and related neurodevelopmental disorders (18, 19, 30, 32, 108, 127, 129–133). The PRS-SA includes 32 items across 5 domains, and each item is rated as typical (0 points), mildly atypical (1 point), or atypical (2 points). The 5 domains include presupposition, discourse management, speech and language behaviors, suprasegmentals, and nonverbal communication. All items were summed to create a PRS-SA total score. In addition, individual items were explored to assess specific pragmatic skills.

The presupposition domain assesses the participant’s ability to understand how information is shared between speakers and how to use language based on the listener’s knowledge in a contextually appropriate manner. The discourse management domain assesses the participant’s ability to maintain a reciprocal conversation. The speech and language behaviors domain assesses the participant’s ability to use speech and language skills that may impact pragmatic success. The suprasegmental domain assesses prosodic features (e.g., speech rate and intonation). The nonverbal communication domain assesses the participant’s ability to use nonverbal behaviors in a social context.

#### PRS-SA reliability

Coders were trained to > 80% reliability on a minimum of 3 training files. Around 50% (N = 65) of the PRS-SA files were coded for reliability, with mean reliability agreement at 81.5%.

##### Conversational speech acts coding

Similar to speech acts for narrative, each utterance was manually coded using a dialog tag set developed for spontaneous spoken speech based on Carletta et al. (126). Speech acts were coded from transcripts using Praat software and examined as a percent of total utterances.

Initiation Statements included initiation of new information that was not directly elicited by the interaction partner. For example, “I did not play a lot of sports in high school” would be captured as an initiation statement if it was not a direct response to the other speaker.

Questions included any questions marked by yes/no, wh-, or intonation (e.g., “Where did you go?”).

Replies included direct responses to a question, and included only the requested information. For example, the reply “I think twice”, in response to, “How many times have you been to Florida?” would be captured as a reply.

Clarifications included utterances that conveyed additional information beyond what was requested. For example, if the examiner asks, “What did you do in New York?”, and the participant responds, “I liked to ice skate”, and then clarifies, “I was never really good at sports”.

Backchannels were characterized as listener feedback to signal accordance (e.g., “yep”, “okay”).

Check-ins were characterized as utterances where a speaker checked attention, agreement, or readiness of the other speaker (e.g., “Does that make sense?”).

Character speech included utterances where the speaker took on a character’s voice (marked in transcript).

Incomplete utterances included abandoned or unintelligible utterances.

#### Conversational speech acts reliability

Transcripts were coded by raters blind to group status. Coders were trained to >80% overall reliability on at least 3 files. Twenty percent (N = 22) of files were double coded for reliability. Reliability was assessed using intraclass correlation coefficients (ICCS) (124). ICCs were greater than .65, indicating moderate to excellent agreement.

##### Linguistic inquiry and word count

Linguistic Inquiry and Word Count (LIWC) was implemented to characterize types of words across narrative and semi-structured conversational contexts for participant speech (134, 135). Data computed using LIWC were examined as a percent of participant word count. Word categories from the LIWC 2022 dictionary include affect (e.g., happy, mad), cognition (e.g., think, believe), and causal words (e.g., because, since). However, the LIWC 2022 dictionary also includes other words in the dictionary that do not map onto theoretical categories. For example, the dictionary for affective words also includes words such as “dork”, “hazy”, and “welcome”. Thus, novel dictionaries were created that were more theoretically in-line with broader word classification categories and tailored to the current study’s language sample and coding approaches.

#### LIWC novel dictionary categories

The full dictionaries can be found in the **Appendix**.

##### Affective States and Behaviors

Simple affective states included words that referenced basic internal emotional states and their synonyms (136–140). For example, happy, sad, angry, afraid, disgust, surprise, and synonyms (e.g., joy, mad). Complex affective states required the ability to reflect on the social environment and were tied to cognitive processes (140–143). Complex affect states were considered culturally-based and may involve layers of simple emotions (140, 141, 144, 145). For example, shame, pride, and guilt. Affective behaviors included words that referenced observable behaviors associated with positive (e.g., hug, smile) or negative (e.g., cry, shudder) emotional valence. Affective states and behaviors captured by LIWC can be found in **Appendix 1a**.

##### Cognitive States and Behaviors

Cognitive states included references to mental states without emotional valence (e.g., know, hope, think). Cognitive behaviors included words that described observable behaviors associated with the mental states of others without clear emotional valence (e.g., escape, follow, hide). Cognitive states and behaviors captured by LIWC can be found in **Appendix 1b**.

Dictionaries were summed to create broader categories. Affective States and Affective Behaviors were summed to create Affective States and Behaviors. Cognitive States and Cognitive Behaviors were summed to create Cognitive States and Behaviors. Words in the Affective States and Behaviors and Cognitive States and Behaviors were summed to create the Affect and Cognition dictionary.

##### Story Elements (Narrative Only)

Dictionary terms included words that mapped onto story elements (e.g., branch, family, antlers) and are found in **Appendix 1c**.

##### Characters (Narrative Only)

All characters were included (e.g., deer, dog, boy) and are described in **Appendix 1c**.

#### Speech act sequences

Specific sequences of speech acts for both narrative and semi-structured conversation were selected a-priori (see below).

##### Narrative speech act sequences

(1) Consecutive on-topic narrative speech: Sequential utterances of a statement, question, character speech, or sound effect. (2) Consecutive off-topic speech: Nonnarrative or thinking utterance followed by another utterance of nonnarrative or thinking language. (3) Consecutive storytelling effect use: Character speech or a sound effect utterance followed by another utterance of character speech or a sound effect.

##### Semi-structured conversational speech act sequences

(1) Non-obligatory response to examiner speech. (2) Backchannel: An examiner utterance (except for questions) followed by a participant backchannel, signaling accordance between speakers (e.g., “yep”, “okay”). (3) Self-elaboration: A participant utterance followed by another participant utterance. (4) Expected speech act following examiner question (i.e., participant reply or clarification). (5) Unexpected speech act following examiner question (i.e., participants’ backchannel or initiation statement).

##### Statistical analysis overview

All analyses, when possible with model parameters, controlled for VIQ, because groups significantly differed and VIQ is known to impact pragmatic language abilities (146–148). While groups differed on word count and number of utterances (see **Table 3**), most variables of interest already accounted for length of conversation or narrative (e.g., examined as percent of word or utterance count), and, thus, length was not included as a covariate. Mazes and reformulations were excluded from word count, but filled pauses were included.

**Table 3:** Word and utterance count by task.

Context	Autistic Males	Autistic Females	Non-autistic Males	Non-autistic Females
	M (SD) range	M (SD) range	M (SD) range	M (SD) range
Word Count				
Narrative^a^	402.27 (167.98) 188-1208	507.16 (248.24) 215-1154	446.88 (221.36) 198-1132	423.00 (150.40) 197-842
Semi-Structured Conversation^a^	2420.30 (1475.98) 369-8420	3662.86 (2277.02) 910-10701	2225.00 (1151.56) 728-5206	2866.12 (1194.99) 977-5684
Utterance Count				
Narrative^a, b^	39.11 (11.74) 25-81	46.92 (17.97) 25-102	38.67 (11.53) 24-62	38.00 (10.11) 24-65
Semi-Structured Conversation^a^	323.31 (130.90) 113-632	429.52 (242.01) 170-1157	283.93 (117.58) 115-643	346.04 (130.72) 169-636

^a^ Significant difference between autistic male and female groups.

^b^ Significant difference between autistic female and non-autistic female groups.

### Analysis plan

All variables were examined for normality of distribution. A series of multivariate analyses of covariance (MANCOVAs) were used to examine the pragmatic language variables separately for narrative and semi-structured conversation. Group differences were examined for diagnosis, sex, and diagnosis*sex interaction effects, and pairwise t-test comparisons using estimated marginal means were examined when the interaction or main effects were significant to determine the direction of effects. Item-level data from the PRS-SA was evaluated using a 4 x 2 or 4 x 3 chi square test, and variables that met the threshold of *p* <.05 were examined using *post-hoc* pairwise comparison 2 x 2 or 2 x 3 chi square tests. Fisher’s exact test was used when 20% or more cells had an expected value of < 5.

To examine distinct sex and diagnostic subgroups across tasks, separate latent profile analyses were used (149) across theoretically relevant, continuous variables for narrative and semi-structured conversational data (see **Table 4**). All variables were z-scored to account for differences in measurement scales and to enhance interpretability. Models specifying one to six profiles were evaluated using an iterative process based on model fit, which was evaluated using the Akaike Information Criterion (AIC), Bayesian Information Criterion (BIC), entropy, class probabilities and sizes, and the Bootstrap Likelihood Ratio Test (BLRT) (149–152) (see **Table 5**). In selecting the optimal model, we also considered the interpretability and size of resulting subgroups. After identifying the best-fitting solution, we conducted follow-up chi square or Fisher’s exact test analyses to explore sex and diagnostic differences in profile membership.

**Table 4:** Measures included in latent profile analysis.

Coding System	Variables
Narrative	
Narrative Quality Coding	Story Structure Cognitive States and Behaviors Causal Explanations
Narrative Speech Acts Coding	Incomplete Utterances
Linguistic Inquiry Word Count (LIWC) Novel Dictionary	Complex Affective States
Narrative Speech Act Sequences	Consecutive on-topic utterances Consecutive use of storytelling strategies
Semi-Structured Conversation	
Pragmatic Rating Scale-School Age (PRS-SA)	Total Score
Conversational Speech Acts Coding	Initiation Statements Questions
Linguistic Inquiry Word Count (LIWC) Novel Dictionary	Complex Affective States Cognitive States and Behaviors
Conversational Speech Act Sequences	Non-Obligatory Responsiveness Backchannel Self-elaboration

**Table 5:** Indices of model fit for latent profile analysis.

Number of Profiles	AIC	BIC	Entropy	Smallest profile proportion	Largest profile proportion	BLRT *p*-value
Narrative						
1	3119.94	3162.64	--	--	--	--
**2**	**3003.10**	**3070.19**	**.96**	**.09**	**.91**	**.01**
3	2869.91	1961.41	.98	.03	.90	.01
4	2887.96	3003.85	.95	.03	.75	.01
5	2879.99	3020.29	.82	.01	.62	.85
6	2804.36	2969.05	.86	.03	.56	.01
Semi-Structured Conversation						
1	3247.80	3295.09	--	--	--	--
2	3175.45	3249.35	.77	.26	.74	.01
3	3139.94	3240.44	.79	.13	.63	.01
**4**	**3129.45**	**3256.55**	**.81**	**.10**	**.47**	**.01**
5	3102.51	3256.21	.79	.09	.37	.04
6	3111.95	3292.25	.81	.05	.36	.01

Bolded values indicate the selected model.

Significance across analyses was set to *p* <.05. Benjamini-Hochberg corrected *p*-values using a false discovery rate of .10 are reported in addition to uncorrected *p*-values (153) in order to capture potentially subtle sex differences.

#### Specific speech act sequence probabilities

**Figure 1:**
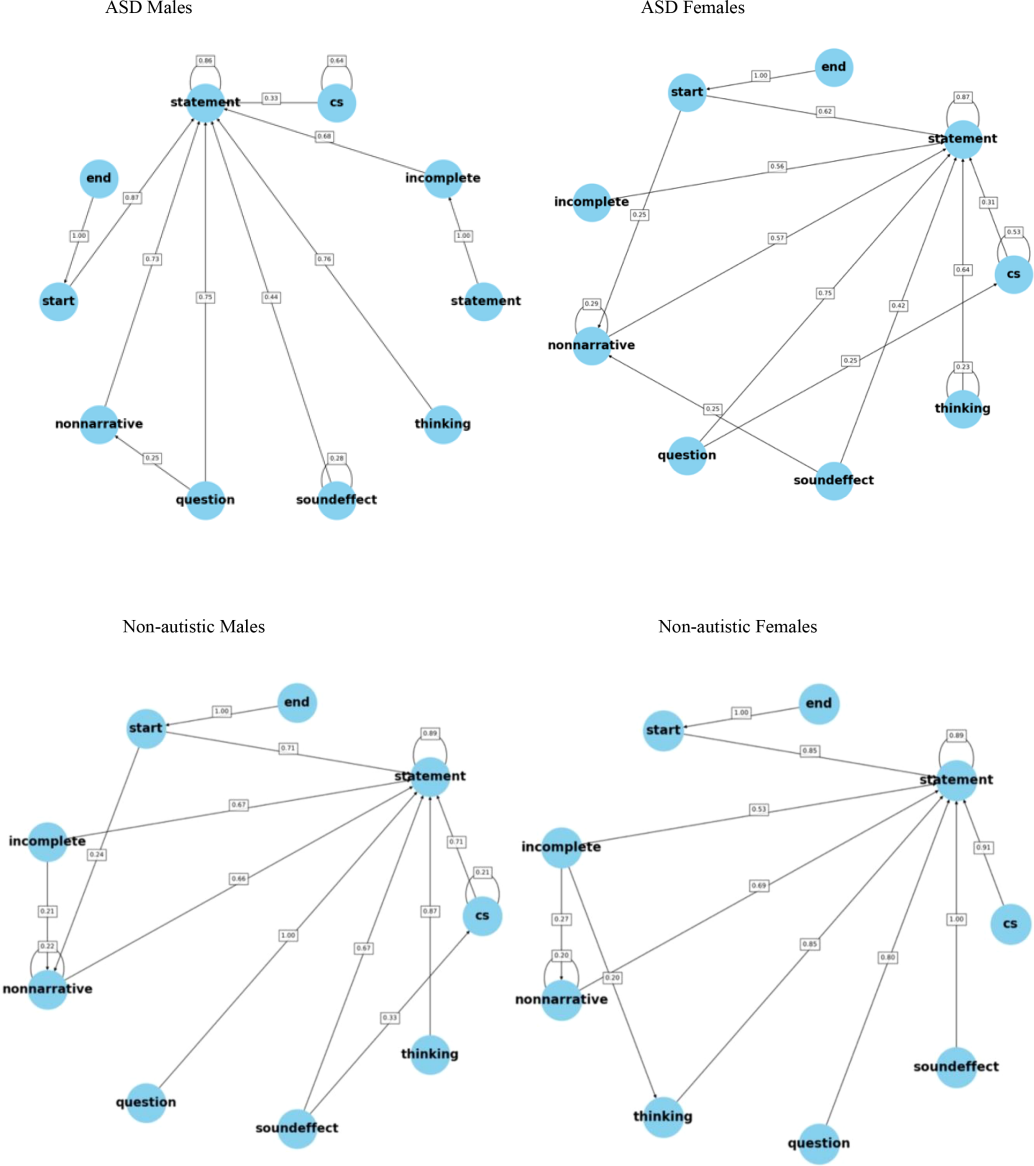
Each figure plots the probability of one speech act following another within each group. Kullback-Leibler divergence models examined overall patterns of sequences of speech acts. The autistic male group marginally differed from the non-autistic male group in the overall pattern of speech act sequences for the narrative. There were no significant differences in the specific patterns of speech acts.

Sequences of speech acts were examined using Markov chain models. Kullback-Leibler divergence models were implemented to calculate the rate of convergence between Markov chains from each group using pairwise comparisons (154). Higher Kullback-Leibler divergence scores indicated greater difference between the distributions of the Markov chains. In the current study, each speech act was plotted as a node (see **Figure 1**).

#### Associations between hand-coded and computational measures

1. For the narrative data, multiple linear regression models were applied to predict hand-coded data from sex, diagnosis, age, verbal IQ, and corresponding word classification domains in all groups combined. When the overall model was significant, significant contributions of specific predictors was assessed. Additionally, partial Pearson correlations assessed associations between the LIWC 2022 and novel dictionaries with VIQ as a covariate and using raw counts of word frequency.
2. Multiple linear regression models were applied to predict Markov chain sequences from the narrative task from sex, diagnosis, age, verbal IQ, and hand-coded domains (story components, descriptions of affect, cognition, and causal explanations) in all groups combined. When the overall model was significant, significant contributions of specific predictors was assessed.
3. Exploratory partial Pearson correlations were applied to assess associations between semi-structured conversational hand-coding (PRS-SA) systems and computational word classification from the conversational task with VIQ applied as a covariate in all groups combined.
4. Multiple linear regression models were applied to predict Markov chain sequences for the semi-structured conversation from sex, diagnosis, age, verbal IQ, and all domains of the PRS-SA in all groups combined. When the overall model was significant, significant contributions of specific predictors was assessed.

## Results

### Hand-coding group and sex differences

#### Narrative quality and speech acts

**Figure 2:**
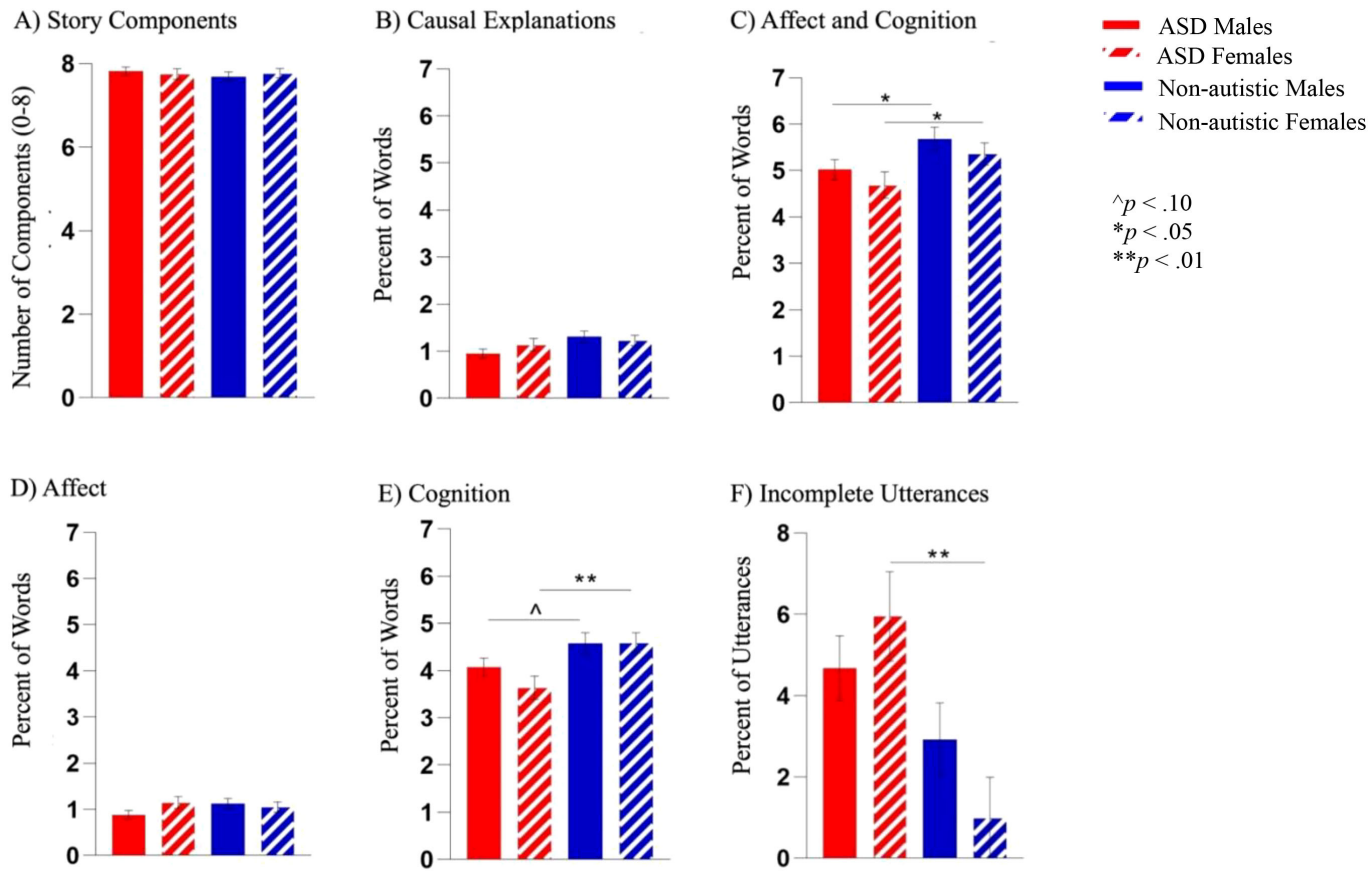
**(A)** Groups did not differ in the number of key story elements described. **(B)** There were no significant differences between groups in the number of descriptions of causal explanations. **(C)** Both autistic groups used fewer descriptions of affect and cognition combined than their same-sex non-autistic comparison group. **(D)** When breaking apart use of descriptions of affect compared to cognition, there were no significant differences across groups in the number of descriptions of affect. **(E)** While both autistic groups used fewer descriptions of *cognition* than their same-sex comparison group, this pattern was only significant between the female groups. **(F)** Autistic females produced more incomplete utterances than non-autistic females, and there were no significant differences between male groups. ^p<.10, *p<.05 and **p<.01.

There were no significant interaction effects (*p*s >.230, adjusted *p*s >.872) (see **Figure 2**). A main effect of diagnosis was found, such that non-autistic groups used more descriptions of cognitive states and behaviors than the autistic groups (F(1, 135) = 10.58, *p* = .001, *ηp^2^* = .073, adjusted *p* = .033). There was a main effect of diagnosis for production of incomplete utterances, where both autistic groups produced more incomplete utterances than non-autistic groups (F(1, 139) = 7.44, *p* = .007, *ηp^2^* = .051, adjusted *p* = .098). No significant differences emerged for other variables (*p*s >.270, adjusted *p*s >.872), including no main effects of sex (*p*s >.063, .352).

#### Pragmatic rating scale-school age

**Figure 3:**
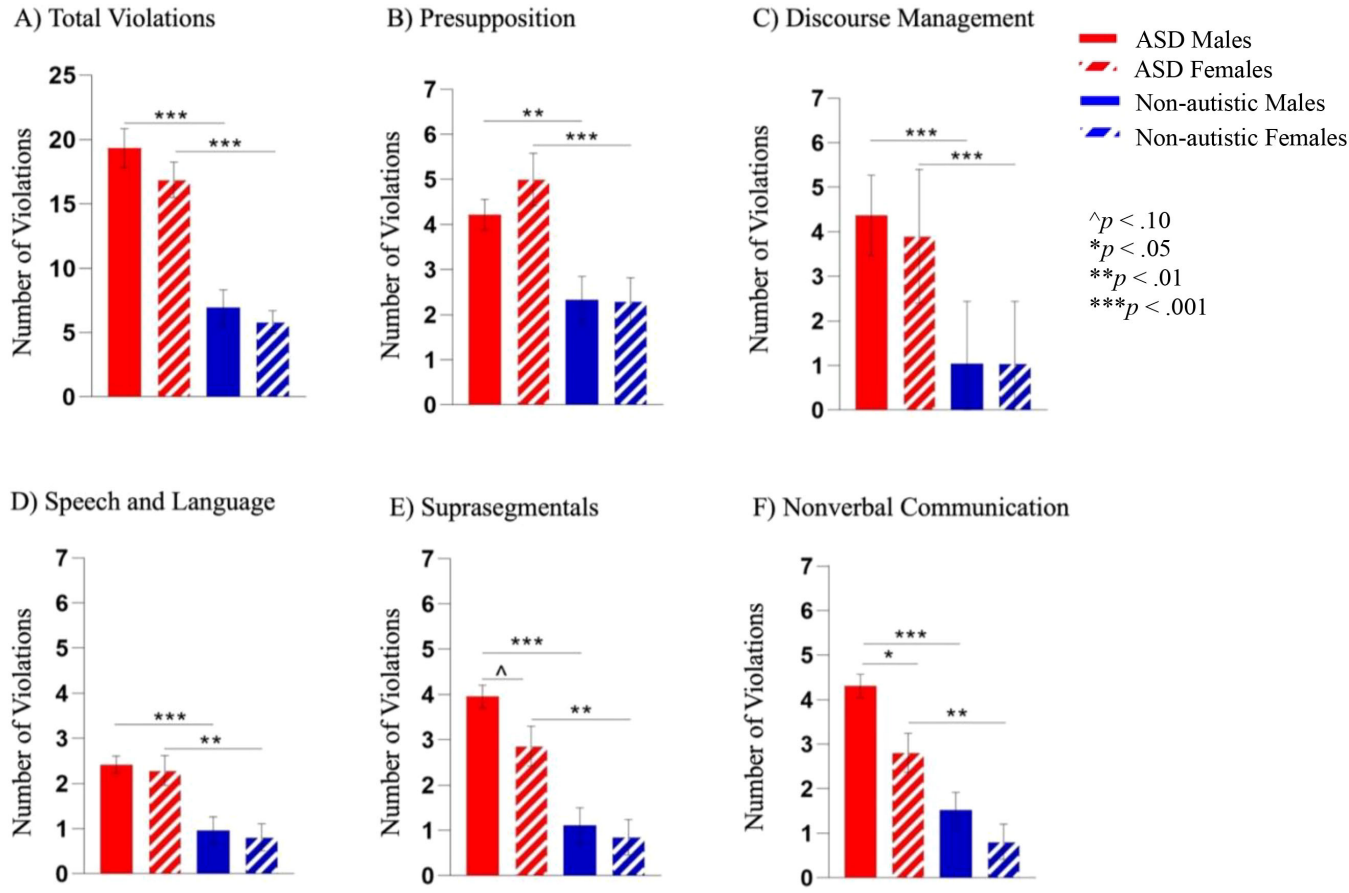
**(A)** Both autistic groups made more violations than the non-autistic groups on the overall PRS-SA score. **(B–D)** Similar to the pattern of overall violations, both autistic groups made more violations than non-autistic groups on the presupposition, discourse management, and speech and language domains of the PRS-SA. **(E, F)** A stepwise pattern emerged where the autistic male group made more violations in the suprasegmental and nonverbal communication domains than the autistic female group, and both autistic groups made more violations in these domains than the non-autistic groups. ^p<.10, *p<.05, **p<.01, and ***p<.001.

There were no significant interaction effects (*p*s >.353, adjusted *p*s >.802) (see **Figure 3**). A main effect of diagnosis revealed that both autistic groups made more violations on the overall PRS-SA as well as all 5 domain scores (presupposition, discourse management, speech and language behaviors, suprasegmentals, nonverbal communication; *p*s <.007, adjusted *p*s <.023). A main effect of sex emerged only in the nonverbal communication domain (F(1, 137) = 7.22, *p* = .008, *ηp^2^* = .050, adjusted *p* = .023), where pairwise comparison of marginal means demonstrated that males committed more violations than females.

See **Table 6** for a summary of item level PRS-SA findings. A greater proportion of individuals in both autistic groups made violations in sharing overly personal information, shifting a conversational topic, interrupting, maintaining reciprocal conversation, using scripted language, modulating speech volume and intonation, producing typical or a range of facial expressions, and maintaining eye contact compared to same-sex non-autistic groups (*p*s <.05, adjusted *p*s <.08). A greater proportion of autistic males made violations in response elaboration, perseveration, grammatical errors, speech rate, production of vegetative sounds, and formulation errors than non-autistic males (*p*s <.008, adjusted *p*s <.023), in speech intonation compared to autistic females (*p* = .017, adjusted *p* = .041), and in gesture violations relative to the autistic female and non-autistic male groups (*p*s <.04, adjusted *p*s <.08). Marginally more autistic females made odd hand mannerisms relative to non-autistic females (*p* = .010, adjusted *p* = .184). Between the autistic groups, a greater proportion of autistic males made violations in redundancy (i.e., shares information previously stated) compared to autistic females (*p* = .016, adjusted *p* = .039), and a greater proportion of autistic females swore relative to autistic males (*p* = .007, adjusted *p* = .022). There were no significant differences in other items (*p*s >.07, adjusted *p*s >.14).

**Table 6:** Pragmatic rating scale- school age.

Domain/item	*p* (overall model)	Significant comparison (greater indicates more violations)
Presupposition		
Overly personal	.006	ASD > Non-autistic group
Swearing	.011	ASD females > ASD males
Overly talkative	.251	--
Overly detailed	.297	--
Insufficient background information	.067	*ASD males > Non-autistic group and ASD females*
Redundant	.005	ASD males > ASD females
Signaling humor	.085	*ASD males > Non-autistic group and ASD females*
Clarification	.200	--
Discourse management		
Topic initiation	.206	--
Topic shifts	.008	ASD > Non-autistic group
Interrupting	.002	ASD > Non-autistic group
Acknowledging	.081	*ASD males > Non-autistic group and ASD females*
Response elaboration	.006	ASD males > Non-autistic males
Perseveration	.001	ASD males > Non-autistic males
Vocal noises	.557	--
Reciprocal conversation quality	<.001	ASD > Non-autistic group
Speech and language behaviors		
Overly formal speech	.361	--
Scripted language	.004	ASD > Non-autistic group
Grammatical errors	.004	ASD males > Non-autistic males
Indistinct speech	.168	--
Suprasegmental speech		
Speech rate	.001	ASD males > Non-autistic males
Pitch/Intonation	<.001	ASD > Non-autistic group; ASD males > ASD females
Loudness	.004	ASD > Non-autistic group
Character speech	.076	*ASD > Non-autistic group*
Language formulation	<.001	ASD males > Non-autistic males
Stuttering/cluttering	.324	--
Nonverbal communication		
Personal space	.365	--
Gestures	<.001	ASD males > ASD females and non-autistic males
Hand mannerisms	<.001	ASD females > Non-autistic females
Facial expressions	<.001	ASD > Non-autistic group
Eye contact		ASD > Non-autistic group
Vegetative sounds	.010	ASD males > Non-autistic males

-- indicates no significant differences.

*P* values for main effects reported. Diagnostic and sex differences were examined when the overall model was significant, and those patterns revealing significant pairwise differences are depicted.

When *p* >.05 and < .10, the directionality is provided in italics.

#### Speech acts

**Figure 4:**
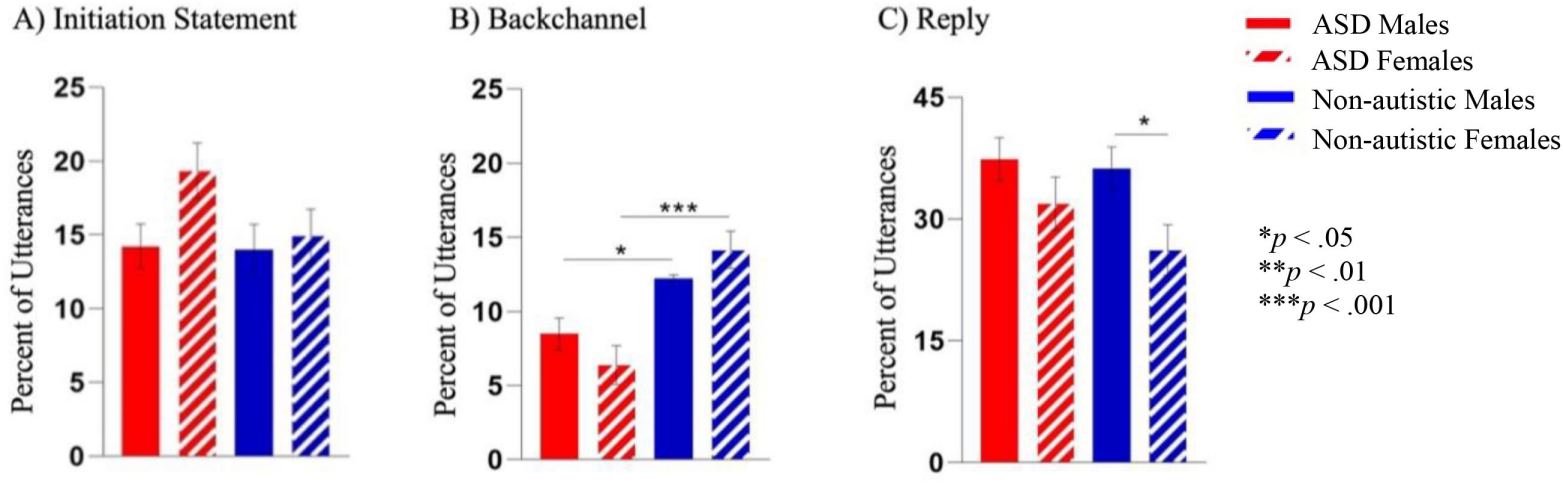
**(A)** There were no differences in the percent of utterances that were initiation statements between groups. **(B)** Both autistic groups used fewer backchannels than the non-autistic groups. **(C)** There was a main effect of sex for replies, with both male groups producing a higher percentage of replies than female groups, with findings only reaching the threshold for significance between non-autistic groups. *p<.05 and ***p<.001.

There were no significant interaction effects (*p*s >.121, adjusted *p*s >.391) (see **Figure 4**). There was a main effect of diagnosis for backchanneling, where pairwise comparison of marginal means demonstrated that autistic groups used less backchanneling than non-autistic groups (F(1, 105) =22.33, *p* <.001, *ηp^2^* = .175, adjusted *p* <.001). Additionally, a main effect of sex emerged for replies (F(1, 105) = 4.52, *p* = .036, *ηp^2^* = .041, adjusted *p* = .252), where pairwise comparisons indicated that male groups had a higher percentage of replies than females (*p* = .020, adjusted *p* = .175). No significant differences emerged in other speech acts (*p*s >.06, adjusted *p*s >.32).

### Computational group and sex differences

#### Narrative word classification (novel LIWC dictionary)

There were no significant interaction effects (*p*s >.177, adjusted *p*s >.530). A main effect emerged for diagnosis, where both autistic groups used fewer words than non-autistic groups (F(1, 151) = 5.77, *p* = .018, *ηp^2^* = .037, adjusted *p* = .030). There were no other significant differences (*p*s >.10, adjusted *p*s >.51).

#### Narrative speech act sequences

Kullback-Leibler divergence models did not detect significant differences between the Markov chains of each group, but there was a marginal difference between autistic and non-autistic males (*p* = .067, adjusted *p* = .526). Comparison of consecutive speech acts did not reveal differences (*p*s >.20, adjusted *p*s >.52).

#### Semi-structured conversation word classification (novel LIWC dictionary)

There were no significant interaction effects (*p*s >.310, adjusted *p*s >.654). A significant main effect of sex demonstrated that females used more complex affective words than males (F(1, 144) = 6.80, *p* = .010, *ηp^2^* = .047, adjusted *p* = .076). There were no other significant differences (*p*s >.15, adjusted *p*s >.43).

#### Semi-structured speech act sequences

**Figure 5:**
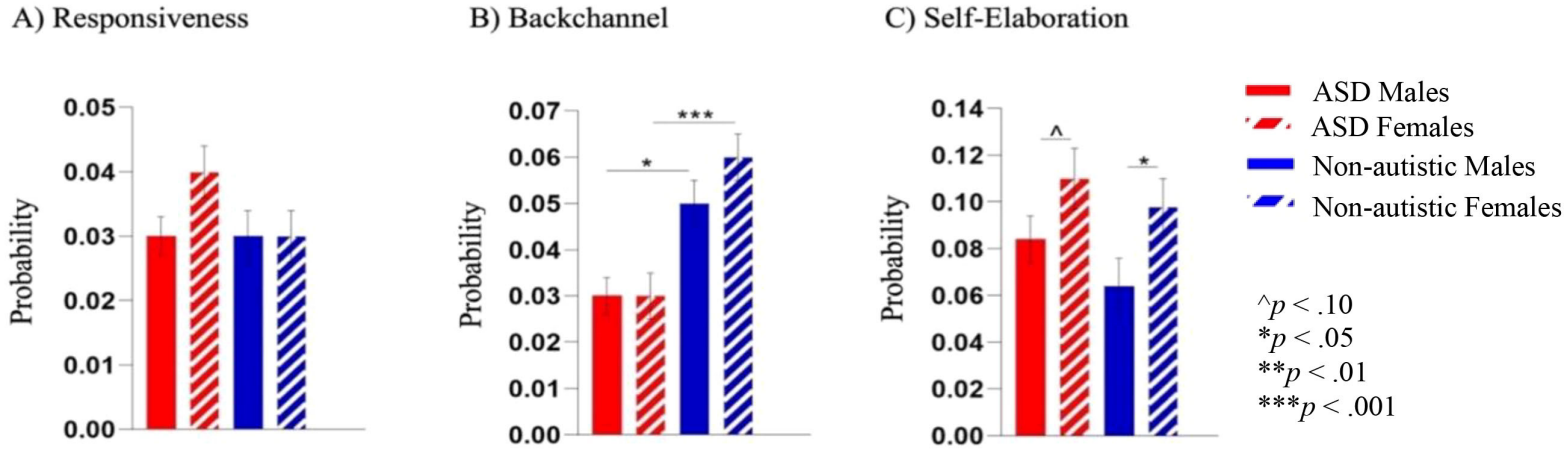
**(A)** There were no significant differences in responsiveness to examiner speech across groups. **(B)** Autistic groups backchanneled less often in response to examiner speech than the non-autistic groups. **(C)** Both female groups were more likely to self-elaborate compared to male groups. ^p<.10, *p<.05, **p<.01, and ***p<.001.

Kullback-Leibler divergence models identified differences in the overall Markov chains between autistic and non-autistic males, and non-autistic males and females (*p*s <.002, adjusted *p*s <.006). There were no significant differences between other group comparisons (*p*s >.50, adjusted *p*s >.82). When examining specific speech act sequences, there were no significant interaction effects (*p*s >.123, adjusted *p*s >.294). A main effect of diagnosis indicated that autistic groups had fewer sequences of backchanneling than the same-sex comparison non-autistic groups (*p*s <.02, adjusted *p*s <.08). Additionally, there was a main effect of sex for sequential utterances (self-elaboration), with pairwise comparison of marginal means demonstrating that females self-elaborated more than males (F(1, 144) = 8.10, *p* = .005, *ηp^2^* = .072, adjusted *p* = .027). See **Figure 5** for specific speech act sequences. There were no other significant differences (*p*s >.13, adjusted *p*s >.29).

### Latent profile analysis

#### Narrative

**Figure 6:**
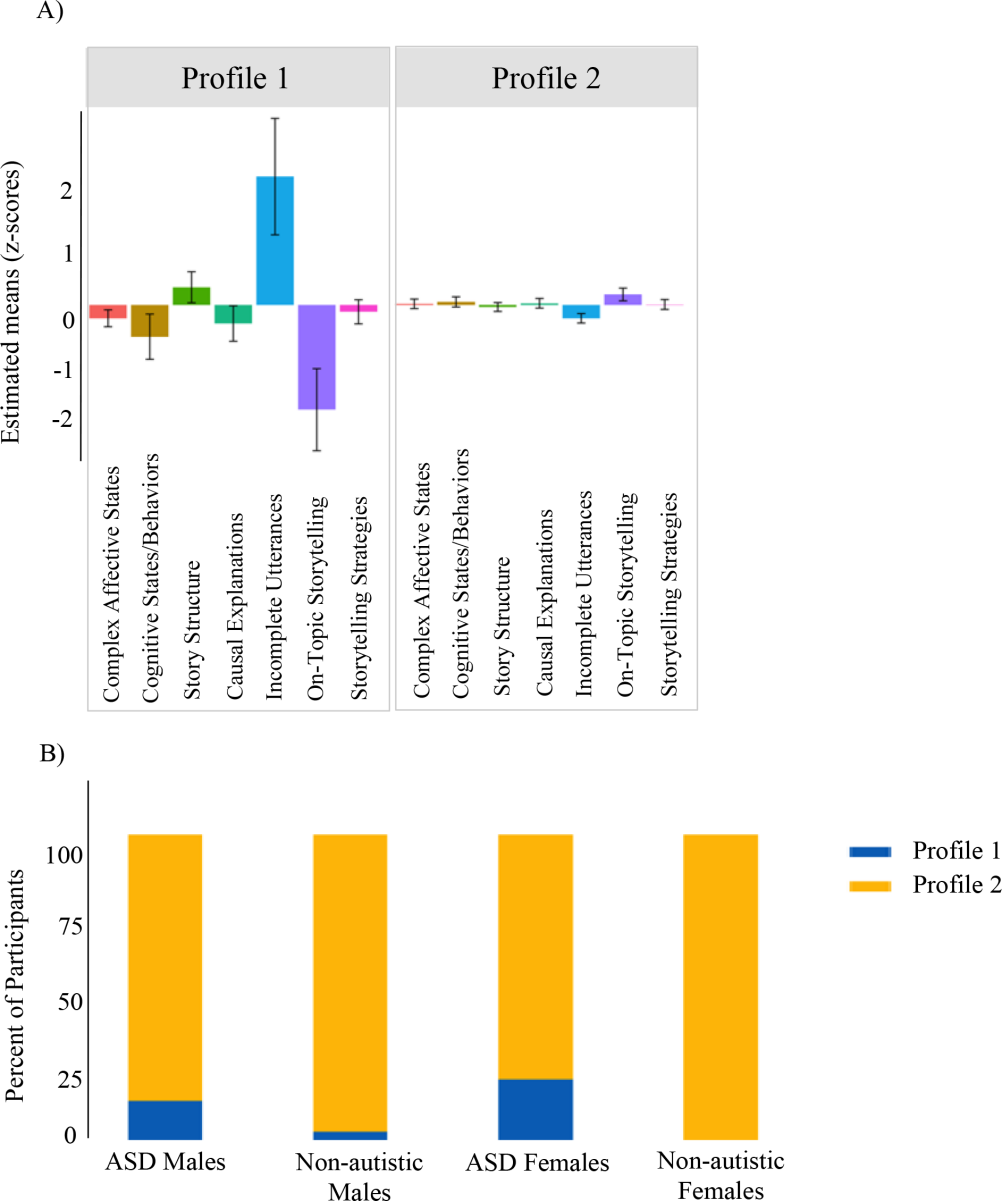
Narrative. **(A)** Latent profile groups reflect patterns across domains. Positive scores reflect greater use of pragmatic behavior. **(B)** Autistic participants were over-represented in Profile 1, while no differences in group membership emerged for Profile 2.

A 2-profile model best represented latent clusters in the narrative data and are presented in **Figure 6**. Profile 1 included individuals with higher rates of incomplete utterances and fewer consistent on-topic utterances. Profile 2 fell around the sample mean. Autistic participants were over-represented in Profile 1 (*p* = .004). For Profile 2, there was no significant differences by sex or diagnosis (*p* = .785).

#### Semi-structured conversation

**Figure 7:**
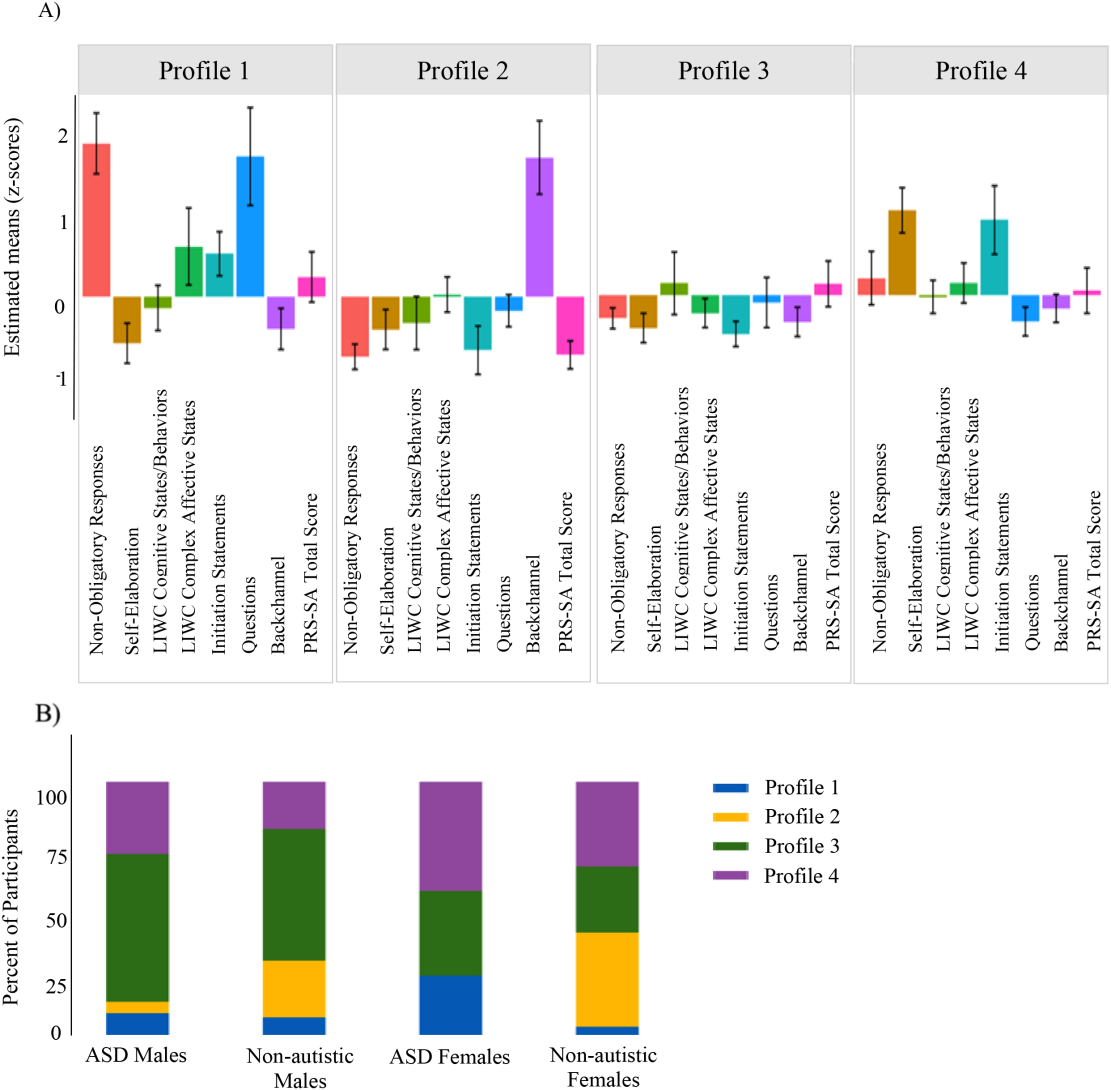
Semi-structured conversation. **(A)** Latent profile groups reflect patterns across domains. Positive scores reflect greater use of pragmatic behavior. **(B)** Sex and group membership analysis found that autistic females were over-represented in Profile 1. Profile 2 was over-represented with non-autistic individuals. Profile 3 was over-represented with autistic males. Profile 4 had higher rates of females.

A 4-profile model best represented latent profiles and are presented in **Figure 7**. Profile 1 included participants who had higher rates of non-obligatory responses to the examiner and asked more questions. This profile was over-represented with autistic females (*p* = .046) relative to the full sample. Profile 2 was characterized by higher rates of backchanneling and was over-represented with non-autistic individuals (*p* = .001). Profile 3 participants clustered around the sample mean, and this profile was over-represented by autistic males (*p* = .046). Profile 4 had higher rates of self-elaboration and topic initiation, and had a higher proportion of females (*p* = .046).

### Associations between hand-coding and computational variables

#### Associations between narrative quality coding and computational word classification

**Figure 8:**
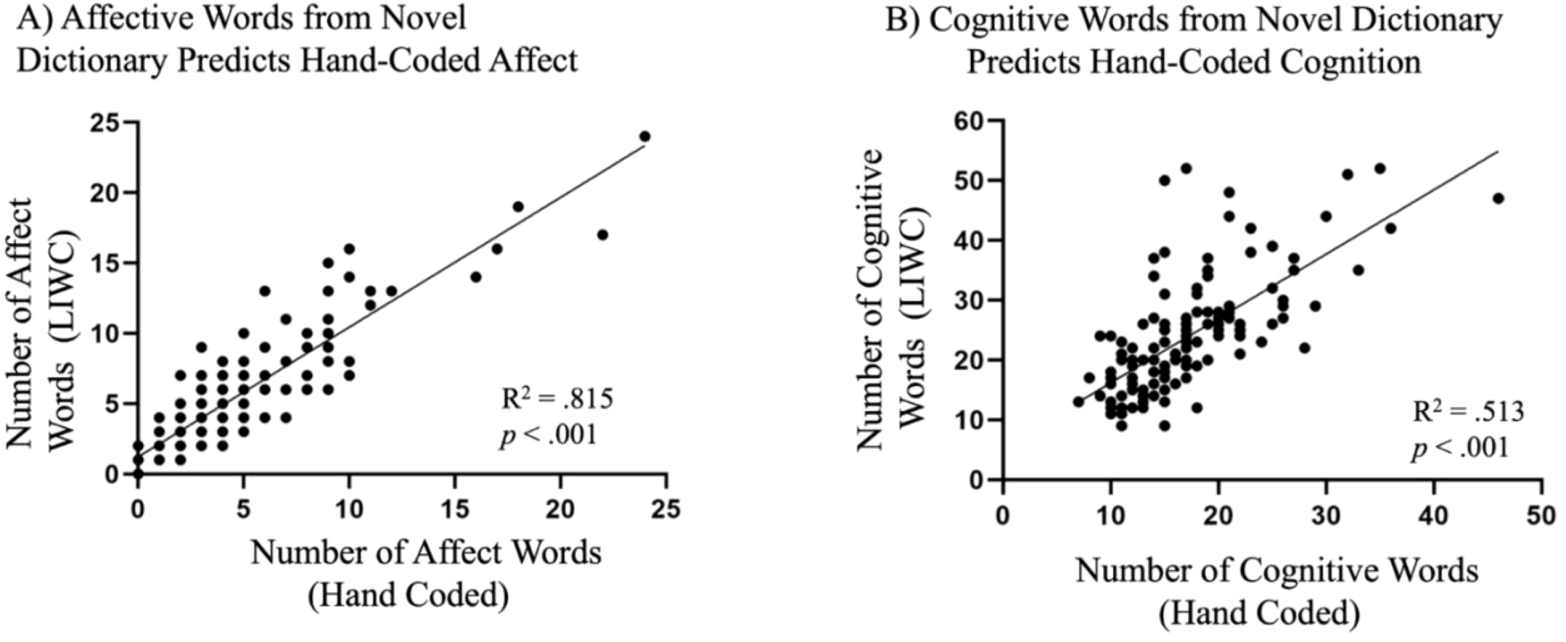
**(A)** Affective words as captured by LIWC significantly predicted hand-coded affect. **(B)** Cognitive words as captured by LIWC significantly predicted hand-coded cognition.

See **Table 7** and **Figure 8** for a summary of the findings. All models significantly predicted hand-coded data in aligned categories (e.g., hand-coded affect predicted LIWC affect; *p*s <.001, adjusted *p*s <.001).

**Table 7:** Means and standard deviation for word categories across dictionaries, narrative.

Coding System	Variable	ASD Male	ASD Female	Non-autistic Male	Non-autistic Female
		M (SD)	M (SD)	M (SD)	M (SD)
Hand Coded Variables	Affective States and Behaviors	3.93 (3.27)	6.29 (6.27)	5.28 (3.74)	4.40 (3.32)
	Cognitive States and Behaviors	15.58 (6.50)	16.17 (5.34)	19.28 (7.05)	18.17 (5.11)
	Causal Explanations	4.24 (3.80)	5.88 (4.27)	6.06 (4.37)	5.46 (3.97)
	Story Components	7.82 (.39)	7.75 (.44)	7.69 (.86)	7.77 (.73)
LIWC 2022 Dictionary	Affect	9.29 (8.64)	11.75 (7.00)	11.25 (7.95)	10.49 (5.18)
	Cognitive Processes	21.73 (15.88)	33.08 (24.34)	27.29 (22.84)	23.83 (13.80)
	Causality	1.44 (2.01)	2.54 (3.23)	1.77 (2.70)	1.83 (1.87)
Novel Dictionary for LIWC	Affective States and Behaviors	5.07 (3.81)	7.29 (5.61)	5.90 (4.50)	5.03 (3.10)
	Cognitive States and Behaviors	21.24 (7.37)	25.83 (12.95)	25.90 (10.43)	24.54 (8.71)
	Story Components	88.29 (24.49)	106.46 (46.96)	101.10 (40.53)	94.91 (33.07)

#### Associations between narrative quality coding and narrative Markov chain sequences

The Markov chain did not predict narrative quality (*p*s >.08, adjusted *p*s >.250).

#### Associations between conversational hand-coding and computational word classification

See **Table 8** for a summary of findings. Use of more affective words was associated with more PRS-SA violations overall and on the speech and language domain (*p*s <.05, adjusted *p*s <.13). More causal language was associated with more pragmatic violations overall, and on the presupposition, speech and language, and suprasegmental domains in particular (*p*s <.05, adjusted *p*s <.17). There were no associations between cognitive states/behaviors and PRS-SA domains (*p*s >.24, adjusted *p*s >.38).

**Table 8:** Associations between semi-structured conversation violations (PRS-SA) and word classification (novel LIWC dictionary).

Variable	Pragmatic Rating Scale, School-Age											
	Total violations		Presupposition		Discourse management		Speech and language		Suprasegmental		Nonverbal communication	
	r	*p*	r	*p*	r	*p*	r	*p*	r	*p*	r	*p*
Affect	.179	.033	.120	.155	.111	.190	.200	**.017**	.147	.083	.100	.239
Cognitive Processes	-.146	.084	-.035	.682	-.104	.218	-.047	.584	-.194	.021	-.156	.065
Causal	.214	.**011**	.303	**<.001**	.026	.760	.278	**<.001**	.170	.044	.081	.340

Bolded values indicate that the finding withstood Benjamini Hochberg corrections.

#### Associations between conversational hand coding (PRS-SA) and Markov chain

Multiple regression models significantly predicted backchanneling (*F*(8, 100) = 3.19, *p* = .003, *R^2^* = .203, adjusted *p* = .017), self-elaboration (*F*(8, 100) = 6.02, *p* <.001, *R^2^* = .271, adjusted *p* <.001), and expected speech acts (*F*(8, 100) = 4.12, *p* <.001, *R^2^* = .248, adjusted *p* = .003), but not responsiveness or unexpected speech acts (*p*s >.09), from PRS-SA domains.

Autism diagnosis significantly predicted less backchanneling (t = -2.79, *p* = .006) and expected speech acts (t = -2.15, *p* = .034; adjusted *p*s <.05). For self-elaboration, significant predictors included sex (t = 2.79, *p* = .006), verbal IQ (t = 2.88, *p* = .005), and the PRSA-SA speech and language (t = -2.76, *p* = .007) and presupposition domains (t = 4.51, *p* <.001; all adjusted *p*s <.02), where females and those with higher verbal IQs had more self-elaboration. More speech and language violations predicted less self-elaboration, while more presupposition violations predicted more self-elaboration in all groups combined. For expected speech acts, PRS-SA domains of discourse management (t = 3.21, *p* = .002), presupposition (t = -2.50, *p* = .014), and suprasegmentals (t = -.2.27, *p* = .025) were also contributors (adjusted *p*s <.08). Discourse management violations predicted more expected speech acts, while more presupposition and suprasegmental violations predicted fewer expected speech acts. Other variables did not significantly contribute to the model (*p*s >.26, adjusted *p*s >.41).

## Discussion

This study applied hand-coding and computational linguistic approaches to characterize potential sex differences in pragmatic language in autistic males and females compared to individuals without autism across narrative and semi-structured conversational contexts. Computational results were interpreted alongside hand-coded data to better understand the meaning and clinical-translational utility of findings. Primary differences emerged in the conversational context, highlighting a subtle and unique presentation in autistic females. Autistic females demonstrated better pragmatic abilities than autistic males in suprasegmental and nonverbal communication domains, though like autistic males, they still showed more pragmatic violations than non-autistic peers. Latent profile analyses further revealed that autistic females were over-represented in certain pragmatic profiles, particularly in initiating social overtures, potentially suggestive of distinct strategies for navigating social interactions. In contrast, autistic males were more likely to cluster in pragmatic profiles reflecting prototypical pragmatic difficulties (e.g., fewer social initiations). Overall, these results highlight how multi-method and context-sensitive approaches clarify sex-related patterns in autism. Specifically, examining pragmatic language across both structured and more naturalistic contexts revealed patterns that would have been obscured in a single context; for example, some sex differences were more apparent in conversational settings that placed higher interpersonal and cognitive demands on the participants. Furthermore, findings help to delineate the strengths and limitations of computational measures relative to hand-coded assessments. In particular, computational approaches offer scalable, reproducible, and nuanced patterns of pragmatic language that, with further refinement, may capture more elements of pragmatic differences than is feasible by hand. Computational tools hold promise for increasing the scale and feasibility of pragmatic language characterization, but continued work is still needed to further refine and optimize the sensitivity of these methods and clarify how computational approaches map onto clinically meaningful pragmatic constructs.

### Narrative

Hand-coded narrative analyses revealed few group differences between autistic groups, which was unexpected in light of prior findings (42, 114, 120, 155–157), and may have been due to the older-aged group of participants in the current study. Autistic groups did produce fewer descriptions of cognition than the non-autistic groups, consistent with the prior literature (42, 158–160), which was primarily driven by differences between autistic and non-autistic females. These findings diverge from prior work reporting greater use of cognitive language in autistic females (69, 78). Notably, both prior studies focused on younger samples (78, ages 8–19; 69, school-aged children) than included in this study. It may be that these sex-specific patterns are evident earlier in development or among individuals with less fluent structural language abilities than the groups studies here. An important further consideration concerns the methods used to capture such language. For instance, Boorse et al. (69) relied on the default LIWC cognitive processes dictionary, whereas the present study employed a task-tailored dictionary specified for cognitive language. The more context-sensitive, targeted approach employed here may therefore have captured important linguistic categories obscured by broader coding categories used in this prior work. Speech acts analysis revealed few group differences, as most utterances were statements. Autistic females did produce more incomplete utterances than non-autistic females, which raises questions about potential underlying mechanisms, such as executive function challenges (e.g., organization, inhibitory control), which have been shown to relate to narrative ability, such that difficulties with inhibition may be associated with more disorganized narratives (161). Markov chains used to explore sequential speech patterns did not reveal differences between groups and failed to predict hand-coded sequences. Given that most utterances in the narratives were statements, this approach was likely too coarse-grained to capture differences in linguistic structures.

This study also used computational word classification approaches, comparing the 2022 LIWC dictionary (134, 135) with a novel, conceptually aligned dictionary tailored to the current study. Autistic females used more complex affective words than other groups, supporting prior findings suggesting sex differences in expressing complex emotions (162). While both dictionaries predicted hand-coded word classification, the novel dictionary outperformed the LIWC 2022 dictionary, yielding higher R^2^ values and tighter clustering around the regression line, indicating closer alignment with the hand-coded data. In addition, the conceptual alignment between the novel dictionary and the category of interest further supports its merit over the broader, less targeted LIWC 2022 dictionary. For example, the inclusion of words such as “hazy” in LIWC 2022’s affective dictionary illustrates a potential mismatch between the default dictionary and the constructs of interest.

### Semi-structured conversation

Consistent with prior work, conversational analyses revealed more robust diagnostic and sex differences than the more structured context (19, 40–42), supporting the value of dynamic, socially interactive settings for identifying subtle pragmatic differences. Across all PRS-SA domains, both autistic groups demonstrated greater overall pragmatic violations than non-autistic peers, consistent with prior literature of widespread pragmatic difficulties in autism (c.f., 16, 18, 21–25, 27–29, 31, 35, 36, 38).

In the suprasegmental and nonverbal communication domains, a stepwise pattern emerged where autistic males showed the most violations, followed by autistic females, then the non-autistic groups. This pattern suggests that autistic females may be more adept at modulating prosody and integrating nonverbal cues, skills central to social communication and diagnostic evaluations, and may contribute to delayed or missed diagnoses. Within suprasegmental features, atypical intonation drove group differences and was observed in nearly 75% of autistic males, compared to only 38% of autistic females, consistent with prior work linking atypical prosody to broader social communication challenges (32, 163–167). In nonverbal domains, more autistic males had atypical gesture use and produced vegetative sounds (e.g., belching), while autistic females showed milder gesture differences but more frequent atypical hand mannerisms. These findings echo literature showing that females may produce more expressive gestures than autistic males (168). Indeed, very few autistic females were rated as having “atypical” gestures (9.5%), while 33.3% were rated as having “mildly atypical” gestures. This contrasts with 38.8% of autistic males with an “atypical” rating and 26.9% with a “mildly atypical” rating. These findings carry clinical relevance, as more typical-appearing gestures and prosody in autistic females could obscure identification of autism, as these are key behaviors rated in autism assessments.

Swearing emerged as a uniquely common violation among autistic females (23.8% of the group) relative to other groups (4.5% of autistic males, 7.4% of non-autistic females, 0% of non-autistic males), possibly reflecting attempts at establishing social connections (169–172), where participants may have perceived swearing as appropriate for peer-to-peer communication, misconstruing the social dynamics of an examiner-examinee relationship and the appropriateness of profanities in this context. In contrast, autistic males showed the most frequent lack of response elaborations, where they did not respond to the interaction partner by adding information as socially expected, aligning with previous findings of reduced conversational reciprocity in autistic individuals (173–175). Although both autistic groups had reduced elaboration on the PRS-SA, autistic females’ violations were milder (i.e., none rated as “atypical”), suggesting a more active conversational style but reduced contingency compared to autistic males. Several pragmatic skills (e.g., inappropriate topic shifts, interrupting) were similar across autistic groups, suggesting that a number of important pragmatic language differences are pervasive across sexes.

Speech act analysis revealed reduced verbal backchanneling among autistic groups compared to same-sex non-autistic groups, consistent with evidence of reduced backchanneling in autistic individuals (173–175). No sex differences emerged, suggesting that this may be a core feature of communication across autistic males and females. Given that backchanneling supports conversational cohesion and mutual engagement (176–178), its reduction in ASD may contribute to downstream social communication difficulties. Interestingly, ratings of acknowledgements from the PRS-SA, which capture both verbal and nonverbal indicators of responsiveness, did not differ significantly between groups, raising the possibility that autistic individuals may rely on nonverbal backchannels, which was not directly assessed in the current study.

Markov chain models employed to capture temporal patterns in the conversational flow revealed that both autistic groups showed reduced probability for backchannel responses, mirroring the hand-coded findings. Additionally, both female groups demonstrated a higher frequency self-elaboration, suggesting possible differences with conversational reciprocity or turn-taking. Although prior research suggests that autistic individuals often exhibit reduced contingency and responsiveness (19, 29, 42, 146, 179), the current speech acts coding system likely lacked the specificity to capture such nuanced differences.

Greater self-elaboration in all groups was associated with increased violations on the PRS-SA presupposition domain, and fewer violations in the speech and language domain, suggesting that increased self-elaboration may be linked to challenges with perspective-taking in conversation, which may contribute to longer speaker turns and difficulties with turn taking. Differences in turn taking and social reciprocity are well documented pragmatic features in autism and have been tied to underlying differences in social cognition (180–187), where it may be difficult for an individual to appreciate when it is appropriate to check in with the other speaker. In contrast, fewer self-elaborations among those with more speech and language violations (e.g., scripted speech, grammatical errors) may be due to difficulties with spontaneous speech production that impacts fluent, extended contributions to conversation.

Computational word classification findings demonstrated that both female groups used more complex affective language than males. However, greater affective word use correlated with increased pragmatic violations across speech and language, suprasegmental, and nonverbal communication domains, suggesting that affective expression does not necessarily equate to communicative success, and also raises questions about the validity of using word categories to predict broader pragmatic language skills.

These findings indicate that computational word classification approaches are most sensitive when they target pragmatic features with clear theoretical and lexical boundaries, such as affective, cognitive, and causal language in the narrative context. In contrast, word classification approaches designed to model discourse-level structure and conversational dynamics showed weaker convergence with hand-coded measures, suggesting reduced sensitivity to pragmatic features that rely heavily on contextual interpretation, interactional timing, and nonverbal cues. Thus, while computational methods offer scalable and efficient tools for indexing certain aspects of pragmatics, they may underrepresent heterogeneity in pragmatic strategies when used in isolation.

### Latent profile analysis

The latent profile analyses provided a complementary framework for interpreting the hand-coded and computational findings. In the narrative context, two distinct profiles emerged. Profile 1 (n=14) was characterized by higher rates of incomplete utterances and fewer consistent on-topic utterances. This smaller group was significantly over-represented by autistic participants, highlighting narrative organization as an area of relative difficulty for this group, consistent with prior literature (e.g., 179, 188). In contrast, Profile 2 (n=142) consisted of the majority of the sample and clustered around the mean, indicating that narrative difficulties in a highly structured context may be common but particularly pronounced in a distinct subgroup.

In the semi-structured conversational context, four distinct groups emerged, reflecting greater variability in pragmatic differences than what was observed in the narrative task. Profile 1 (n=14) included individuals who produced higher rates of non-obligatory responses and asked more questions, features that may reflect attempts to maintain conversational reciprocity. This group was over-represented by autistic females, aligning with evidence suggesting that autistic females may exhibit more socially-oriented pragmatic behaviors, even in the context of broader social communication challenges (52–57). Profile 2 (n=19) was defined by frequent backchanneling and was over-represented by non-autistic individuals, consistent with literature highlighting more backchanneling in non-autistic groups (173–175). Profile 3, the largest group (n=67), was clustered near the sample mean across all pragmatic features, and was over-represented by autistic males. The presence of a “mean-level” group dominated by autistic males may reflect a more prototypical presentation of ASD-related pragmatic difficulties. Finally, Profile 4 (n=42) was characterized by greater rates of self-elaboration and topic initiation and was over-represented by females across diagnostic groups. These features may reflect a more self-directed communication style, consistent with findings that unequal topic initiation between speakers can be a marker of conversational dominance (189). The contrast between Profiles 1 and 4, both of which were over-represented by autistic females, underscores the heterogeneity within this group. Profile 1 reflected a pattern specific to autistic females, characterized by increased verbal engagement, while Profile 4 captured broader female-specific features across diagnostic groups, in which participants tended to actively guide or steer the interaction.

### Study strengths, limitations, future directions, and clinical implications

A primary strength of the study was the focus on pragmatic language in autistic females, an understudied population in neurodevelopmental linguistics research. The use of both narrative and semi-structured conversational contexts provided a richer understanding of how pragmatic language skills manifest across varying social demands. Another notable strength was the use of multiple methodologies, combining traditional hand-coding techniques with computational linguistic tools. Directly comparing these methods allowed for the evaluation of the validity and limitations of automated linguistic tools. The latent profile and convergence analysis provided complementary insights into how computational approaches might meaningfully index particular pragmatic features. At the same time, these tools could obscure heterogeneity when used in isolation, highlighting the importance of multi-method approaches are key for capturing the diversity of pragmatic language profiles in autism.

One important limitation concerns the lack of assessment of masking, where individuals conceal or minimize autistic traits to appear more socially “typical” (11–15). Autistic females, in particular, are more likely to engage in masking behaviors, often through subtle linguistic strategies (11, 190–192). Anxiety, which is highly co-occurring in autism and especially in autistic females, may also interact with these masking behaviors and influence social communication (193–198). Whereas the current study necessarily focused on verbally fluent autistic individuals, extending this research to include those with lower verbal abilities is critical, as pragmatic language challenges may manifest differently (199). Future research should also focus on narrower age ranges, such as adolescence, to more precisely characterize developmental patterns in pragmatic language, as pragmatic expectations change across development (16, 200). It will also be important to extend the current findings to include gender identity in addition to sex assigned at birth. Gender identity is a multifaceted construct that may not align with assigned sex, and autistic individuals are more likely to identify as gender diverse compared to the general population (201), particularly for autistic females (202, 203).

One potentially important clinical implication of findings stems from results showing that gesture use and intonation [pragmatic domains considered in an ASD diagnosis (39)] appear to be areas of relative strength for autistic females. These strengths may obscure diagnostic identification, particularly when clinicians rely on overt markers of social communication difficulties. Clinicians should be mindful that the absence of traditional pragmatic deficits does not rule out autism, particularly in females. Beyond individual clinical practice, these findings underscore the need for updated gold-standard instruments and scoring approaches that are sensitive to female-specific presentations of autism, including modifications to item selection, scoring criteria, or algorithmic thresholds to improve diagnostic accuracy. Although the computational methods used in this study may not yet be feasible in most clinical settings, they hold promise for augmenting traditional assessments, with further refinement. With continued development, automated tools could support more efficient identification of pragmatic language differences in autism and other neurodevelopmental conditions, especially in time-constrained environments.

## Data Availability

The raw data supporting the conclusions of this article will be made available by the authors, without undue reservation.
