# Supplementary material for: A multi-method phenotypic study of sex differences in pragmatic language in autism
Source: Front Psychiatry. 2026 Apr 22;17:1759436. doi: 10.3389/fpsyt.2026.1759436 (PMC13147202; doi:10.3389/fpsyt.2026.1759436)
Supplement: Supplementary file 1 [file SupplementaryFile1.docx]

Appendices

***Appendix 1a. Affective States and Behaviors.***

**Affective States.**

| Afraid^S^ | Devastat*^S^ | Happi*^S^ | Mad*^S^ | Satisf*^S^ |
| --- | --- | --- | --- | --- |
| Anger^S^ | Disappoint*^C^ | Happy^S^ | Merry*^S^ | Scare*^S^ |
| Angr*^S^ | Disgust*^S^ | Hopeless* ^S^ | Miser*^S^ | Scaring*^S^ |
| Annoy*^C^ | Dislik*^S^ | Horror*^S^ | Miss*^S^ | Scary*^S^ |
| Anticipat*^C^ | Elate* ^S^ | Humiliat*^C^ | Nervous*^S^ | Shame*^C^ |
| Asham*^C^ | Embarass*^C^ | Infuriat*^S^ | OK*^S^ | Shock*^S^ |
| Bliss* ^S^ | Envy*^C^ | Interest*^S^ | Panic*^S^ | Sorrow*^S^ |
| Care*^S^ | Exasperat*^C^ | Irate^S^ | Petrif*^S^ | Surpris*^S^ |
| Caring^S^ | Excit*^S^ | Irk*^C^ | Pride*^C^ | Terrified^S^ |
| Cheer*^S^ | Fear*^S^ | Irritat*^S^ | Proud*^C^ | Terrify*^S^ |
| Concern*^C^ | Fright*^S^ | Jealous*^C^ | Regret*^C^ | Trust*^C^ |
| Content*^S^ | Frustrat*^C^ | Jolly*^S^ | Relief*^C^ | Uneas*^S^ |
| Crank*^C^ | Furious*^S^ | Jovial*^S^ | Reliev*^C^ | Unhappy*^S^ |
| Cross^S^ | Glee*^S^ | Joy*^S^ | Repuls*^S^ | Upset*^S^ |
| Delight*^S^ | Grief^S^ | Loath*^S^ | Resent*^C^ | Worri*^C^ |
| Depress*^S^ | Grieve^S^ | Lonel*^C^ | Revolt*^S^ | Worry*^C^ |
| Despair*^S^ | Guilt*^C^ | Lov*^S^ | Sad*^S^ |  |
| *Indicates any letters following the * would be captured, such as in conjugation.  ^S^Simple Affective State  ^C^Complex Affective State | | | | |

**Affective Behaviors.**

| Bit* | Cried | Kiss* | Smil* |
| --- | --- | --- | --- |
| Cry* | Frown* | Laugh* | Tear* |
| Cries | Hug* | Lick* | Whimper* |

*Indicates any letters following the * would be captured, such as in conjugation.

***Appendix 1b. Cognitive States and Behaviors.***

**Cognitive States.**

| Amaze* | Engross* | Imagin* | Inquis* | Think* |
| --- | --- | --- | --- | --- |
| Believ* | Expect* | Interest* | Inquir* | Tire* |
| Belief* | Guess* | Inquis* | Learn* | Tiring |
| Decid* | Idea* | Inquir* | Oblivious* | Want* |
| Distract* | Fun* | Know* | Preoccup* | Asleep |
| Confus* | Hope* | Know* | Question* | Sleep* |
| Contemplat* | Hoping | Imagin* | Realize* | Slept* |
| Curio* | Know* | Interest* | Realizing | Wonder* |

*Indicates any letters following the * would be captured, such as in conjugation.

**Cognitive Behaviors.**

| Accus* | Comfort* | Investigat* | Push* | Throw* |
| --- | --- | --- | --- | --- |
| Adopt* | Continu* | Knock* | Rescu* | Tipto* |
| Attack* | Crept | Lie* | Run* | Trip* |
| Atten* | Creep* | Lying* | Sav* | Try* |
| Bat* | Dream* | Look* | Search* | Trie* |
| Bother* | Dump* | Mess* | Shelter* | Watch* |
| Call* | Escap* | Observ* | Shoo* | Wak* |
| Captur* | Follow* | Peek* | Sneak* | Woke |
| Carry* | Help* | Peer* | Sniff* |  |
| Catch* | Hid* | Pester* | Swat* |  |
| Chas* | Hover* | Play* | Swear* |  |
| Check* | Introduc* | Pretend* | Refuge* |  |

*Indicates any letters following the * would be captured, such as in conjugation.

***Appendix 1c. Story Elements (Narrative Only).***

Story Elements includes words in both the story events and characters tables below. Please note that words in the story events category also appear in other dictionaries, as this category pulls from all types of word categories that commonly appear in descriptions of the key story elements.

**Story Events (Narrative Only)**

| Adopt* | Child* | Flower | Lick* | Sneak* |
| --- | --- | --- | --- | --- |
| Air | Cliff | Follow* | Lift* | Splash |
| Antler* | Climb | Found | Lily* | Stick* |
| Asleep* | Cloth* | Hand | Listen* | Sting* |
| Attack* | Couple | Head* | Log | Stop |
| Babies | Cover* | Hear | Mom* | Stuck |
| Baby | Dad* | Hold* | Missing | Tipto* |
| Bed* | Disturb* | Hole* | Moon* | Tree* |
| Bit* | Dream* | Home | Night* | Wak* |
| Branch* | Ear | Hug* | Parent* | Water |
| Bother* | Escap* | Husband | Play* | Wav* |
| Boot* | Fall* | Gone | Pond | Wet |
| Bump* | Family | Goodbye | Quiet | Wife |
| Bye | Fell* | Ground* | Rock* | Window* |
| Call* | Fell* | Jar* | Run* | Woke |
| Carry* | Find | Kid* | Sit* |  |
| Chas* | Finger | Knock* | Shh |  |
| Check* | Floor* | Ledge | Sleep* |  |

*Indicates any letters following the * would be captured, such as in conjugation.

**Characters (Narrative Only)**

| Animal* | Buck* | Frog* | Mole* | Toad* |
| --- | --- | --- | --- | --- |
| Antelope* | Chipmunk* | Hawk* | Moose* | Wasp* |
| Beaver* | Creature* | Groundhog* | Owl* | Woodchuck* |
| Bee* | Deer* | Gopher* | Reindeer* |  |
| Bird* | Dog* | Honeybee* | Rodent* |  |
| Boy* | Elk* | Hornet* | Squirrel* |  |

*Indicates any letters following the * would be captured, such as in conjugation.
